# Explaining specific taxes management and use in the health sector: a qualitative study

**DOI:** 10.1186/s12913-022-08556-4

**Published:** 2022-09-30

**Authors:** Mahdi Kooshkebaghi, Sara Emamgholipour, Hossein Dargahi

**Affiliations:** 1 Department of Health Management and Economics, School of Public Health, Tehran University of Medical Sciences, Tehran, Iran; 2 Department of Health Management and Economics, School of Public Health, Health Information Management Research Center, Tehran University of Medical Sciences, Tehran, Iran

**Keywords:** Specific taxes, Harmful goods, Sin tax, Value-added taxes, Green taxes, Management

## Abstract

**Background and aim:**

Being the major source of revenue and essential economic tool for policymakers to improve public health, taxes contribute to government spending on the development of health care facilities and services. Given the financial challenges facing the health sector together with the public health issues that affect each society, placing specific taxes on some goods, services, and activities can be effective in this regard. The study aims to explain the various dimensions of specific taxes in the health sector and management of these resources in order to achieve the health system goals.

**Materials and methods:**

This study with a qualitative research design was conducted using semi-structured interviews with open-ended questions in 2020–2021. In total, 38 managers, policymakers, economists, key experts, and other individuals, as informants, were interviewed. Purposive and snowball with maximum variation was also employed. As well, content analysis was utilized to shed light on the data. The transcribed interviews were further imported into MAXQDA for extracting and classifying the relevant codes.

**Findings:**

In this study, 5 main themes and 23 subthemes were labeled. The main themes accordingly included “Objectives and Conditions of Specific Health Taxes”, “Earmarked Taxes”, “Taxes on Goods and Measures of Harmful to Health”, “Value-Added Taxes”, and “Green Taxes”.

**Discussion and conclusion:**

Considering the specific taxes in the health sector, i.e., taxes on goods and measures of harmful to health, value-added taxes, and green taxes, all taxation and pricing policies need to take account of the effects as well as the advantages and disadvantages of types of taxes, a country’s economic structure, the conditions of industries and manufacturing enterprises, cultural aspects in society, and peoples’ socioeconomic status.

## Introduction

As a sustainable and stable financial resource that is less affected by political and economic events, taxation plays an important role in financing government measures, including health programs [1, 2]. As well, an efficient and fair tax system is vital for the continuity of governments’ activities [3, 4]. By levering taxes, governments can also finance spending and take further steps toward meeting three main goals, including: economic stability, resource allocation, and revenue distribution. For example, they can reduce demands for the production of some specific goods, particularly unnecessary and even harmful ones, by putting taxes on them. In this way, governments can influence consumer behavior by taxation [5]. Therefore, among the many sources of government revenues, taxes are thus the most acceptable and logical ones [6, 7].

After oil, taxes in Iran are the most important source of government revenue. In recent years, the share of tax revenues in total government revenues has increased significantly. However, the tax system in Iran suffers from problems such as lack of comprehensive tax information system, complexity of the tax system, low executive capacity and weak economic structure [8]. In most countries, tax revenues are the main source of financing and development of health systems. For every $ 100 more per capita in tax revenue each year, about $ 10 per capita in health spending increases [2]. But Iran’s position in this index among the countries of the world is not appropriate [9]. Although taxes are known as important economic tools for policymakers to improve public health [10], at the same time, the taxes on harmful goods and measures in Iran is not very significant and effective. For example, the total share of tobacco taxes as a percentage of retail price is only 21.7% [11]. However, according to the World Health Organization (WHO), this amount should be 70% to be effective [12].

On the other hand, How to provide sustainable financial resources is further today one of the challenges facing health policymakers, which is increasing day by day following the growing needs for public services in different countries, on the one hand, and financial constraints, on the other hand [13]. Tax revenues can be used for health care expenditures or to offset other sources of revenue for governments [14, 15].

The governments purpose of specific taxes is primarily to discourage the consumption of unhealthy products, and then to generate revenue [16]. The use of taxes for a healthier lifestyle is of interest to scientific and academic centers [17], and the WHO has recommended the use of these financial instruments [18–20]. Taxes and subsidies are considered in health policies, because unhealthy products are not economically affordable by increasing the price, and as a result, the incentive to consume these products reduce, or by decreasing the price of healthier products, their consumption becomes economically affordable and increases [21].

Taxation brings major benefits to the development of the health sector; for example, establishing a health system based on tax revenues can guarantee equal and equitable access to health care facilities and services by all segments of society. Taxes can be similarly utilized as a lever to control harmful goods or as an incentive to improve health systems in different countries [22].

Evidence also shows that taxes levied on harmful goods as well as rising prices have thus far reduced consumption rates so that, for every 10% growth in the prices of such products, their total consumption has dropped to 4 and 8% in developed and developing countries, respectively [23].

In a study in Vietnam in 2018, increasing taxes on tobacco, including cigarettes, had significantly diminished the adverse effects of the use of these substances, and prevented the loss of $57 billion of the country’s resources [24]. A 2016 survey in South Africa had similarly demonstrated that taxation, especially Value-Added Taxes (VATs), on sugary drinks, could prevent about 72,000 deaths and 550,000 years of healthy living lost due to stroke. Such policies could also reduce health expenses by $5 billion [25].

If the main goal of health tax policy is to reduce the consumption of unhealthy goods, the price of products should increase by 20% or more. However, when taxes change health behaviors, the predictability of the revenue flow is reduced [26]. Due to the social, economic and health harms associated with harmful products, attention has been paid to the ability of taxes to raise the costs of manufacturing, distribution, retail or consumption of these products, which can reduce their consumption [27].

Theoretically, taxes on polluting industries and activities along with environmental standards have thus far provided a suitable tool for controlling and compensating for external costs caused by pollution from various organizations and factories, as well as reducing pollution and emissions to an appropriate level in a steady manner [28]. In a study in Iran (2017) the results showed, health expenses due to reduced air pollution had significantly dropped following the rise in green tax rates [29]. It was also shown in an article in the UK (2011) that taxation could have a significant effect on the consumption of environmentally polluting goods by increasing the prices of goods [30].

In addition to having significant health benefits, tax policies can be used to increase public health budgets. These policies encourage healthy behaviors by modifying incentives to prevent disease and making better lifestyle choices, which have significant effects for expenditures of public health and private sector [31].

Given the insufficient funds allocated to providing health care facilities and services and even the ignorance about tax revenues, especially specific taxes, in many countries, amending the existing laws to limit tax evasion and develop tax incentives can be among the tactics to reduce financial problems and prevent the incidence of diseases in society [32]. Therefore, the present study examines the obstacles and problems, as well as the strategies for absorption, allocation, and use of specific health taxes.

The health system should provide the necessary financial resources to provide facilities related to the health of various people in society, especially the needy [33], and specific taxes such as taxes on harmful goods and measures, VATs and green taxes, are the most important and sustainable of these resources.

In order to increase the effectiveness of specific health taxes, legislation should be done in a proper way. Legislation in Iran is mainly done by the parliament. The plans and bills, after the approval of the Parliament, must be approved by the Constitutional Council in order to become law. Next, the president signs the enacted law and provides it to relevant organizations and ministries for implementation [34].

Given the financial challenges facing the health sector and the public health issues that affect this society, imposing specific taxes on many goods, services, and activities makes it possible to deal with such problems in this regard. So far, there has been no research in Iran on specific taxes related to the health sector and even no attempt in the other countries examining all the important aspects of specific taxes in the health sector, so the present study was significant in this sense. The objectives of this study are examining and explaining various aspects of specific taxes in the health sector, including VATs, taxes on harmful goods and measures, and green (or environmental) taxes, and the management and use of tax resources related to the health sector. As a result, the challenges standing in front of the health sector, particularly the problems of financing and distribution of financial resources could be firstly reduced, and secondly, public health status could be improved by tackling some difficulties in the healthcare sector, especially through minimizing the incidence of diseases in society and providing proper access to health care facilities and services for all. Policymakers, lawmakers, managers, economists, experts and researchers in the fields of health, taxation, general sector economics, and environment are the most important audiences of this study. In this research, questions were asked about the goals, effects, problems and strategies for absorption and allocation each of the specific taxes related to health, and how to use these taxes.

## Materials and methods

### Study design

The present case study with a qualitative research design was conducted in 2020–2021 in Iran. This research was exploratory and explanatory and its study units, were specific taxes related to health. The study was performed with a qualitative approach so as to gain a comprehensive, deep, and rich information of the subject. In addition to individual interviews, the data in the present study were collected through a review of documents and archival data to make the research results more valid. Upstream laws and documents, such as tax laws and development program laws, as well as reports from organizations such as the Ministry of Health and Medical Education, Iranian National Tax Administration (INTA), the Central Bank, and the Parliament Research Center, were among the most important archival documents and data that were examined. This type of documentation and data, provided researchers with a good perspective and background, especially for designing interview questions, conducting and coding interviews, and analyzing findings.

According to the criteria of COREQ checklist [35], in this study, in order to provide a complete and transparent report and improve the rigor, comprehensiveness and credibility of interviews, important aspects of the research including context of the study, expression of the problem, study methods, findings, analysis and interpretation, Were presented.

### Participants and setting

The statistical population consisted of managers, deputies, policymakers, economists, key experts, and other individuals, as informants, from INTA, the Ministry of Health, the Ministry of Economic Affairs and Finance, the Plan and Budget Organization, Department of Environment, members of the parliament (MPs), as well as managers, professors, and key experts working in top research centers and universities across Iran.

The inclusion criteria in this study were holding at least a master’s degree or a medical doctorate (MD), having an administrative background or educational and research activities in the field of economics and taxation, as well as showing their willingness to attend the interviews. In total, 38 managers, deputies, policymakers, economists, and key experts were interviewed upon obtaining their consent. The information about the interviewees’ place of employment and demographic information, are illustrated in Tables 1 and 2. Purposive and snowball sampling with maximum variation was also employed. Accordingly, the interviewees were selected by the researcher as well as suggestions by previous interviewees, because they needed to either have a certain feature of a phenomenon or rich information about a particular case [36]. Convenience sampling was used to recruit participants. The interviews also continued until saturation was reached with regard to the collected data.

**Table 1:** Interviewees’ place of the employment

Organization	Count	Percentage
Tehran University of Medical Sciences	7	18.42
Iran University of Medical Sciences	1	2.63
Shahid Beheshti University of Medical Sciences	1	2.63
University of Tehran	2	5.26
Allameh Tabataba’i University	1	2.63
Islamic Parliament of Iran	3	7.90
Iran National Tax Administration	12	31.58
Ministry of Health and Medical Education	7	18.42
Ministry of Economic Affairs and Finance	1	2.63
Plan and Budget Organization	2	5.26
Department of Environment	1	2.63
Total	38	100

**Table 2:** Demographic information of the interviewees

Variable/ Sub-variable	Count	Percentage
Gender		
Male	36	94.74
Female	2	5.26
Age (Year)		
30–40	9	23.68
41–50	10	26.32
51–60	15	39.47
Above 60	4	10.53
Work experience (Year)		
5–10	3	7.90
11–20	13	34.21
21–30	13	34.21
Above 30	9	23.68
Level of education		
Masters	13	34.21
Medical	5	13.16
PhD	20	52.63

Due to the research conducted in Iran and the available samples, as well as the difficulty of interviewing experts from other countries, all interviewees were selected from Iran. Of course, some of the interviewees in this study have executive backgrounds or cooperation with international economic and health organizations.

### Data collection

To develop the interview guide and the related questions, existing rules, standards, and guidelines, research background, along with expert opinions were utilized. In order to determine the validity and assure the significance of the questions from the respondents’ points of view, three pilot interviews were also conducted with the members from the statistical population who were not among the selected interviewees, the type and order of the questions were then identified, and the questions that were unintelligible to the interviewees were revised. Ultimately, the final guide and interview questions were developed. Naturally, the topics that were more closely related to the individuals’ expertise, responsibilities, and knowledge were questioned in each interview and evaluated in detail.

The most important questions was asked from the interviewees in this study, include the following:

- Do you think the current rate of taxes on harmful products such as tobacco and cigarettes, sugary foodstuff, fatty foodstuff, soft drinks and other carbonated beverages, etc. is appropriate? If not, why?
- How can we impose a higher VAT or a separate tax on the products and practices that have greater negative effects on public health and give the revenue to the healthcare system?
- Are the current green tax rates, especially for highly polluting industries, appropriate and deterrent enough for preventing or reducing environmental damages and whether they contribute to public health? If not, why?
- Upon receiving appropriate tax revenues, what should be the priorities of the Health sector in using the received resources? (If possible separately for taxes on harmful goods and measures, VAT and green tax)

The interviews were conducted in a semi-structured manner with open-ended questions; in this way, first, general questions were addressed to start the interview, and then, the interview process was oriented based on the respondents’ answers. The interviews were further conducted based on having a prior appointment and choosing a location by the interviewees over a period of 9 months. Thus, after coordination, most of the interviews were conducted at the respondents’ workplace and some were completed by phone calls as wished by them. The topics of the interview sessions were also recorded with the permission of the interviewees and then transcribed verbatim. The interviews were also transcribed immediately after the end of each session in order to become aware of the data saturation time and increase their accuracy. Out of 38 interviews, 32 and 6 cases were conducted in person and by phone calls, respectively.

Considering that all the interviewees were from Iran, the interviews were conducted in Persian. After transcribed, categorizing and coding the interviews, the final text was translated into English by a reputable translation agency. Then, the translated text was also checked by the researchers of this study.

### Trustworthiness of data

The accuracy, precision, and robustness of the data were further determined based on the four criteria of credibility, transferability, dependability, and confirmability [37]. To ensure the credibility of the data, member checks, peer feedback, allocation of enough time to data collection, and researchers’ long-term involvement were used. The transferability of the data was also provided through interviews with different informants from various departments and a rich description of the data. To ensure the dependability criterion, the study process was submitted to quality consultants and they approved the study results after some reviews. These quality consultants were two university professors, who are not part of the research team. In order to meet confirmability, indicating the stability of the data, along with observing the researchers’ impartiality, external reviews were utilized in the form of supplementary comments by colleagues and review of Manuscripts. Thus, the interviews, coding, and the extraction of the main themes and subthemes were fulfilled by two members of the research team who did not have any conflict of interest with the research subject.

### Data analysis

Content analysis as a qualitative method was further exploited to analyze the data and determine the existence of certain words and concepts to better summarize, describe, and interpret the data [38]. After conducting the interviews, their transcriptions were read several times to gain a general understanding. In addition, the transcribed interviews were sent to the interviewees if they wished to add or subtract some issues while confirming them. Finally, the transcribed interviews were entered into MAXQDA (ver. 12) for classification and the initial codes were extracted. Then, the final codes and themes were obtained with the cooperation and valuable comments of the interviewees and members of the research team, and at last, the codes were grouped into main themes and subthemes.

### Ethical consideration

This article approved by Tehran University of Medical Sciences, Tehran, Iran, with the ethics code: IR.TUMS.SPH.REC.1398.332. Prior to the interviews, the research objectives along with the arrangements required to maintain the confidentiality of the data were presented orally to all interviewees, and written consent was acquired.

## Results

According to the transcribed interviews and a review of archival documents and data, five main themes and 23 subthemes were identified in this study. The themes and related quotes are listed in Table 3, at the end of the results. The main themes including “Objectives and Conditions of Specific Health Taxes”, “Earmarked Taxes”, “Taxes on Goods and Measures of Harmful to Health”, “VATs”, and “Green Taxes”.

**Table 3:** Themes, Sub-themes and related quotes

Themes	Sub-themes	Quotation	The Workplace of the interviewees
**Objectives and Conditions of Specific Health Taxes**	Health Taxation Requirements	“From what sources should we initially collect the taxes? We must also clearly identify the right and proper ways to collect them. Economically, we divide the resources according to the amount of money that can go to the health sector or in terms of the impact it can produce.”	Ministry of Health
	Health Provision, Maintenance, and Promotion	“Taxes are assumed as control tools that, can be also effective in providing, maintaining, and promoting health. In order to meet the priority of health over treatment, we must thus pay much more attention to the laws that can lead to the reduction of medical expenses and the promotion of public health.”	Ministry of Health
	Sustainable Financing	“Sustainability seems to be more evident in the sectors, practices, and goods that are highly related to health, so the ones that are imposing higher costs on the health sector need to be taxed more in order to be more sustainable.”	Iran National Tax Administration
	Changing Health-Related Behavior in Society	“Health status naturally improves when people’s behavior changes. Taxes are also assumed as a tool to change and improve behavior in various ways, and consequently control consumption.”	Tehran University of Medical Sciences
	Reforming the Health Sector Structure	“Taxes give rise to the economic sector reforms and even regulations in the health sector.”	Ministry of Health
**Earmarked Taxes**	Type of Taxes	“Earmarked taxes like value-added ones are very important in the health sector. The production and sale of some goods and services definitely affect the health system, and here, taxes must be levied. Another form of taxation should be also imposed on businesses due to environmental pollution.”	Ministry of Health
	Advantages	“Specific taxes improve financial capacity, better mobilize resources for the health sector, establishing a logical link between the resources allocated and the specific programs and better compliance and coping with budget problems and constraints.”	Islamic Parliament of Iran
	Disadvantages and Problems	“Resource allocation in a specific manner can make it difficult to allocate to other sectors. On the other hand, some influential groups may abuse their power to get more resources.”	Ministry of Economic
	Requirements and Conditions for Absorption, Allocation, and Use of Tax Resources	“First of all, the government must be careful enough that the resources allocated in the health sector are effective in terms of the priorities of the country. Second, resources should not be wasted by merely allocating them specifically to the Ministry of Health and Medical Education. The third point is that supervisory bodies such as the parliament and the judicial system should properly oversee the spending of credits in the health sector.”	Iran University of Medical Sciences
	Health Tax Fund	“The tax resources spent for public health should be definitely collected in a fund with a single trustee. Accordingly, resources can be allocated to the relevant organizations and ministries by good planning, and there should be much care about where these resources are allocated.”	Islamic Parliament of Iran
**Taxes on Goods and Measures of Harmful to Health**	Taxation Objectives, Effects, and Requirements	“Taxing harmful goods can have two reasons and purposes: first, rising taxes and prices of such products will reduce their consumption rates, and this will promote public health, second, taxes levied on these goods can be a sustainable source of funding for the health system.”	Ministry of Health
	Problems related to Taxes on Goods and Measures of Harmful to Health	“The growing consumption of some goods that are harmful to health and the increase in the prevalence rate of diseases and their consequent damage are in part due to tax policies and incorrect pricing. Weaknesses in the enforcement of regulatory and control laws and poor tax structures can be two important reasons in this respect.”	Iran University of Medical Sciences
	Dealing with Taxes on Goods and Measures of Harmful to Health	“When we fail to reduce the consumption rate of harmful goods, we need to at least define a risk for them, that is, to make them more difficult to achieve, I mean, to make them so expensive that, firstly, people do not tend to buy them, and secondly, if they go for them and it is proven that a disease has been caused by consuming such products, we will give their share by consumers.”	Tehran University of Medical Sciences
	Tax Use and Health Sector Share	“Taxes on harmful goods can be used to help industries and manufacturing enterprises to produce better and healthier products, or even acquire healthier raw materials at better and lower prices. They can be further used for the treatment of some complications and related diseases.”	Islamic Parliament of Iran
**Value-Added Taxes (VATs)**	Taxation Objectives and Effects	“The three effects of value-added taxes, can be sustainable and reliable financing for the country and the health system, influencing people’s consumption rates, so that health-related costs are also minimized with rising prices and reduced consumption of harmful goods and actions, and improving production processes and modernizing production lines.”	Islamic Parliament of Iran
	Problems and Difficulties related to VATs	“The main reason for the non-administration of value-added taxes is the lack of infrastructure and no transparency so that when a product is manufactured and circulated in different chains, we cannot observe that chain.”	Iran National Tax Administration
	VAT Rates	“The rates of value-added taxes should be determined in such a way that the country’s production and employment capacities are taken into account, people’s consumption rates are reduced, and in fact, consumption patterns, on the one hand, and production lines and processes, on the other hand, are corrected in a positive direction.”	Islamic Parliament of Iran
	Tax Use and Health Sector Share	“There has to be a direct link between the numbers of services the agencies provide and the tax and tolls they receive.”	Ministry of Health
**Green Taxes**	Taxation Objectives and Effects	“If we want to point at the effects and benefits of green taxes, we can refer to improvements in the environment, economic productivity and ability to increase public revenues, transparency and promoting public health.”	University of Tehran
	Problems and Difficulties related to Environmental Degradation	“The costs of reducing pollution are very high. If you want to remove the pollution from a factory and fix it, it costs a fortune; I mean, you have to buy some equipment, hire several technicians, and redesign production lines.”	Iran National Tax Administration
	Separation of Green Taxes from VATs	“Placing green taxes, under the tax clause or commentary, is no longer the right thing to do, because there is so much literature and space for green taxes, and it can be so effective, to address them separately.”	Ministry of Health
	Dealing with Pollution and Environmental Degradation	“We need to offset this pollution and reduce the costs, but not just by taking environmental measures, but creating green space, updating equipment and using modern technology, and replacing technology with, for example, those with less energy use and pollution.”	Iran National Tax Administration
	Tax Use and Healthcare Sector Share	“We should give this money a good place that can have two characteristics: one is being more cost-effective, that is, it produces more social welfare, health care facilities and services, and consequences, and the other is the issue of justice, which is also important wherein there is a need.”	Tehran University of Medical Sciences

### Objectives and conditions of specific health taxes

#### Health taxation requirements

Considering the economic situation and indicators of the country (inflation, downturn, economic growth, unemployment, etc.), cultural situation, beliefs of the people, attention to the positive and negative effects of health taxes on production and consumption, the existence of goods and healthy alternative services, were important requirements mentioned by the interviewees. Another the most central requirements for specific taxes in the health sector is to separate and group the cases and examples affecting public health. Another requirement is that such taxes should not be seen from only one perspective; this means that in the taxation, various objectives and perspectives such as health promotion, sustainable financing and improving consumer behavior, should be considered.

#### Health provision, maintenance, and promotion

Health provision, maintenance, and promotion are the main goals of placing taxes on goods, services, and activities that affect health. Taxation should be thus a tool that seriously takes account of health-promoting aspects, and from this perspective, prevention becomes of utmost importance. Taxes can help provision, maintenance and promotion of health in a variety of ways, including: reducing the consumption of harmful goods and services, reducing the production of pollutants and damaging the environment, developing and upgrading health facilities and infrastructure, developing education and attracting expert and sufficient personnel in the health sector, modernization of equipment and improvement of production lines, expanding the production of healthy goods and creating a culture and raising public awareness.

#### Sustainable financing

Adequate and sustainable financing in the health system using taxes can accordingly have its own requirements. First, transparency in tax resources and related budgets is a necessity, second, all sectors that directly or indirectly affect the health sector are either taxed or some tax incentives are considered for them, third, the relevant tax bases need to be strengthened and taken more seriously, fourth, the cooperation and consensus of various experts and organizations should be used for sustainable financing practices and the way they are fulfilled, and fifth, other sustainable financing strategies, along with taxes, such as insurance should be also taken into account.

#### Changing health-related behavior in society

Taxes can modify individual or group behaviors in society. They can also improve consumption patterns. Most of the interviewees accordingly highlighted the need to enact tax laws and policies in this regard. This issue could thus have positive impacts on consumers and even production.

#### Reforming the health sector structure

According to a number of interviewees, taxes related to the health sector could help reform the existing structures in the health system. Making changes in the financing structures and health-oriented approaches could be thus among the important effects of taxes on the health sector infrastructure.

### Earmarked taxes

#### Type of taxes

According to the interviewees, the examples of specific health taxes included taxes on products with impacts on health, VATs, and green taxes.

#### Advantages

As stated by the interviewees, specific health taxes could bring some benefits such as revenue generation, sustainability, health promotion, accountability, effective implementation of health care priorities, overcoming challenges facing financial management system, resource cost determination, increased cost efficiency, supply and demand balancing, and inhibitory effects.

#### Disadvantages and problems

Specific health taxes can have their own disadvantages and problems such as corruption and irregularities in the economic structure, lower budget flexibility, waste of resources, difficulty in monitoring and evaluation, conflicts with budget flexibility, reduced government ownership of resources, and shortages in resources for other sectors, loss of integrity of health care policies, the preference of individual and organizational issues over national interests, as well as disrupted budget integrity and comprehensiveness.

#### Requirements and conditions for absorption, allocation, and use of tax resources

Modeling in line with existing conditions and localization, prioritized allocation and consumption of resources according to the facts, transparent spending, monitoring and follow-up, executive guarantee system, attention to time framework and specific purpose for health tax allocation, tax imposition effects, as well as the use of health taxes were among the most important requirements and conditions mentioned by the interviewees in this study.

Tax resources should be collected and then allocated according to some correct criteria, particularly those for taxes on products with impacts on health. Such allocations must be also accurate and timely. Some interviewees did not consider the allocation of more resources to the health system to be logical, and some reflected on the allocation of tax resources to the health sector as a type of investment.

#### Health tax fund

One of the suggestions raised by some interviewees was to establish a fund called the Health Tax Fund. Specific tax revenues should be thus collected in this fund and allocated to organizations and bodies related to the health sector under strict supervision, and with regard to logical and precise principles. The reason for the importance of the Health Tax Fund, is the integration of specific tax revenues, allocation based on performance and needs, transparency in the using of tax resources, and regular and accurate monitoring of these resources.

Other suggestions were mainly related to the direct allocation of tax revenues to relevant ministries and organizations. Another suggestion of the interviewees, was to allocate specific tax revenues in the form of general budgets of organizations and ministries, and not to need for direct allocation of resources.

### Taxes on goods and measures of harmful to health

#### Taxation objectives, effects, and requirements

The interviewees here stated that the government needed to correctly determine the objectives behind taxing goods and services. An effective tax rate for goods and services could not be also set until the taxation objectives had become clear and good tax efficiency was achieved. The effects of taxes on harmful goods could be also positive, according to most interviewees. Moreover, individuals’ financial capability in the income groups and the effects of imposing taxes on consumption during different periods needed to be taken into account.

Also, the examples of taxes on products with impacts on health must be identified in a correct manner and according to the reality of society. In addition, grading such products and dealing with them according to degrees of damage is a very important issue. Most of the interviewees accordingly mentioned the elasticity of harmful goods and services in some way. Reactions to tax filing could also vary greatly depending on the type of elasticity.

#### Problems related to taxes on goods and measures of harmful to health

The complexity of the tax system, tax laws with no deterrent effects, poor policies, inadequate enforcement guarantees, challenges in manufacturing products, current standards and prices, smuggling, unforeseen individual and social behaviors, and self-interest in some individuals and groups were among the problems related to taxes on goods and measures harmful to health.

#### Dealing with taxes on goods and measures of harmful to health

Most of the interviewees stated that in order to deal with these goods and measures, the best strategy is to use tax and non-tax solutions at the same time. One way to combat products with impacts on health is the correct administration of taxes on them. Taxes should be such that their effects on reducing consumption rates and the incidence of diseases are tangible. Also, creating a culture and raising public awareness about harmful goods and actions, paying attention to all factors affecting demand and supply, determining suitable alternatives, as well as subsidizing healthy products, are among the most important non-tax solutions.

#### Tax use and health sector share

Most of the interviewees reflected on the uses of taxes placed on goods and measures of harmful to health for developing preventive measures, reducing out-of-pocket payments, improving healthcare services, and increasing public access to appropriate health care facilities and services.

### Value-added taxes (VATs)

#### Taxation objectives and effects

VATs are one of the most important sources for sustainable financing of the health system. Given their unique nature, such taxes can thus have significant effects on economic stability and financial regulation compared with other types of taxes.

On the other hand, the goods and services on which VATs can be imposed and allocated to health-promoting activities must be properly identified.

#### Problems and difficulties related to VATs

According to the interviewees, inadequate infrastructure, the same tax rates for most goods, the possible effects of inflation, pressure on consumers, and no transparency in tax revenues were the most important issues related to VATs, which could have a significant impact on the allocation of resources to the health sector.

#### VAT rates

Most of the interviewees stated that different VAT rates could be set on goods and services that affect health, and even reduce them on useful and healthy products, or exempt them.

#### Tax use and health sector share

In allocating resources, all ministries and agencies should be viewed with the same and equitable view. Increasing VATs and allocating them to the health sector should be also disciplined and reasonable. The activities of some organizations can be further health-promoting. As well, the uses of VAT resources should be more closely monitored and their effects need to be identified.

### Green taxes

#### Taxation objectives and effects

Green taxes should be filed according to different conditions such as elasticity in the market and consumer preferences. The main objective of administering green taxes must be providing access to the necessary information and proper monitoring. As well, the mechanisms to collect such taxes and their proper spending, as well as their deterrence effects must be evaluated.

Also, as shown in the opinions of a number of interviewees, the examples of pollution and environmental degradation, as well as their extents, should be accurately identified in order to increase the effectiveness of green taxes, and then taxation should be implemented step-by-step manner according to the degree of pollution and damage.

#### Problems and difficulties related to environmental degradation

According to the interviewees in this study, the high costs of equipment and processing modernization, the low costs of environmental degradation and economic inefficiency for violators, no willpower in industry ownership to reduce pollution, non-deterrence effects of taxes and fines, non-compliance with some important standards in environmental areas, as well as no expiration-date compliance for various industrial and productive goods, were challenges facing environmental pollution and health impacts.

#### Separation of green taxes from VATs

Approximately two-thirds of the interviewees in this study considered the independence of green taxes and their separation from VATs as an essential. At the present, green tax is part of VAT. Given the effects of environmental pollution on public health and the increase disease, mortality and financial costs caused by these pollution, green taxes need to be a separate tax base. This will bring more serious attention to environmental pollution and its effects, as well as increase the effectiveness of green taxes.

#### Dealing with pollution and environmental degradation

Most polluting industries and manufacturing enterprises were required to take compensatory measures so that part of their harmful activities would make up for the environment. In order to prevent or reduce environmental degradation, proper legislation and policies must be developed and implemented. Among the important tasks to minimize health expenses is to provide environmental attachments for industrial projects. According to several interviewees, the use of the authority and capacity of universities and knowledge-based companies as well as their financial support should be respected.

#### Tax use and healthcare sector share

Allocating green tax resources to beneficial environmental measures and reducing environmental degradation, according to a number of interviewees, was the most important usage of green taxes. A percentage of green tax resources must thus go to the Environment Fund. Upgrading technology in the biggest polluting factories and industrial units, renewing training programs, building a culture, as well as using empowerment and monitoring activities are among the most important measures to diminish environmental degradation. It is also necessary to spend a percentage of green tax resources on health-promoting measures.

## Discussion

Specific health taxes contribute to sustainable financing in the health sector and improve public health by changing habits and modifying lifestyle patterns. All ministries and agencies must thus play a significant role in absorbing, allocating, and making the best use of specific health taxes, so the required legislation, proper planning and implementation, as well as periodic monitoring must be done in this regard. According to the interviews in this study, five main themes were established, including “objectives and conditions of specific health taxes”, “earmarked taxes”, “taxes on goods and measures of harmful to health”, “VATs”, and “green taxes”.

The study results accordingly revealed that taxes were one of the most important sources of revenue that could be exploited in the health sector. In Nadri and Khodabakhshi study [39], taxes revenues had similarly led to significant positive effects on health expenditures in Iran. Thus, through taxes we can help improve health indicators in society and solve the problems facing the health system, especially providing health care facilities and services and meeting medical expenses.

A systematic review of the research on health taxes indicated that policy actors need to be clear about the primary goal of any health tax and frame the tax accordingly [27]. Sustainable financing, changing health-related behavior in society, providing, maintaining, and promoting health, and reforming the health sector structure were also among the most important goals of specific health taxes addressed in this study.

Earmarked taxes include the separation of all or part of tax resources, in order to be allocated to a specific purpose in the health sector. Many countries use dedicate resources, to mobilize resources and finance the health sector. According to the World Health Organization, more than 80 countries use earmarked resource [40]. Earmarked taxes have requirements that Modeling in line with existing conditions and localization, prioritized allocation and consumption of resources according to the facts and transparent spending, are the most important of these requirements. Also, performance accountability, accurate and continuous monitoring and follow-up and executive guarantee system, are very important for the establishment of specific taxes.

The resources for specific health taxes should be thus allocated in a balanced manner to all organizations and agencies involved in the health system, and unquestionably, the authority and supervision of the Ministry of Health and Medical Education in the health sector should be maintained. Establishing a fund called the Health Tax Fund was thus one of the suggestions mentioned in this study.

In recent years, many countries have imposed new or higher taxes on a broader array of harmful goods, or have structured taxes in new ways with the aim of increasing the cost of manufacturing, distributing and consuming these goods. For example, since 2010, countries such as Denmark, Hungary, Finland, France, Mexico and the United Kingdom, have imposed taxes on the sale of unhealthy foods and beverages [41].

The effects of taxes on harmful goods such as cigarettes, sugars, high-fat substances, and alcohol, according to the results of this study, could be positive but some considerations needed to be observed. Imposing taxes could typically increase the prices of harmful products and decrease their consumption rates, resulting in fewer health threats. In a 2017 study in Australia, obesity in the general population had fallen by 1.96% with a 20% tax on sugary drinks. In addition to boosting economic productivity, these changes had also saved 35,000 years of life and reduced $425 million in health expenses [42]. Another research (2008) in the United States had further found constructive evidence that higher taxes had moderated smoking by older adults, especially those with less education and living in low-income families [43].

Evidence shows that tobacco taxation policies play an important role in achieving significant health outcomes in low- and middle-income countries (LMICs), especially among the poor, as well as help reduce poverty [44–49]. In 2012, Powell et al. had shown that pricing tools could be considered and evaluated as potential policy tools to pursue health risk factors [50], which were consistent with the findings of the present study.

In taxation, individuals’ financial capability in the income groups and the effects of imposing taxes on consumption rates in different periods, as well as the elasticity of goods should be taken into account. On the other hand, the examples of taxes on goods and measures harmful to health must be identified correctly and according to the reality of society. Excise taxes generally result in higher prices for consumers, reducing demand for taxed products. The size of the reduction and whom prices most affect depend on consumers’ price elasticity of demand [16]. According to the WHO and the United Nations, taxes on harmful goods should be increased to reduce the consumption of these goods [51, 52]. This approach is important, because consumption patterns are influenced by purchasing prices [53].

Sin taxes represent one way of raising revenue and, through that, creating fiscal space (FS) [54]. These taxes have significant benefits, especially on health consequences [55, 56]. According to Partyka et al. [57], sin tax, if implemented properly, could help change consumer behavior and thus positively affect public health. Sin taxes also can reduce the consumption of unhealthy goods and generate more revenue [58]. Evidence has shown that the application of these taxes can have a significant effect on consumption patterns and population welfare [59]. The present study correspondingly showed that specific taxes in the health sector, could lead to sustainable financing and improve public health by increasing the prices of goods and services.

Given their unique nature, VATs could also have significant effects on economic stability and financial regulation compared with other taxes. The effects of such taxes, especially on health-threatening goods and industries could include sustainable financing for the health system and reducing the consumption rates of harmful products and actions, thereby moderating health expenses as well as improving production processes and renovation of production lines. In this sense, Keen and Lockwood [60] had further found that the application of VATs had boosted productivity and efficiency in most of the countries that have adopted them.

Based on the results of the present study, different VAT rates could be set on goods and services affecting health. In setting the taxes and determining the rates of VATs, the reform of consumption patterns and production processes should be considered. In Seyed Nourani et al. [61], it had been also suggested to reflect on two optimal VAT rates according to the type of goods.

As demonstrated in the present study, inadequate infrastructure and non-transparency of tax revenues were among the most important problems related to VATs, which could have a significant impact on the allocation of resources to the health sector. In Ziyai [62], the complexity of National Tax Administration, Insufficient information, inappropriate software, and inadequate training of tax officials could hinder the optimal implementation of the VAT system. In the research by Mobini [63], three categories of barriers, i.e., cultural, administrative, and structural, had been further identified as the main barriers to the establishment of the VAT system.

Regarding the allocation and use of VAT resources, all ministries and agencies should be viewed with the same and equitable perspective. Increasing VATs and allocating them to the health sector should be thus disciplined and reasonable. Improving the infrastructure of the health sector and meeting the health needs have deprived and less privileged areas, are the most important uses of VAT.

Another important issue was that green taxes should be filed according to different conditions such as elasticity in the market and consumer preferences. Goal setting for green taxes accordingly requires access to the necessary information and data, proper monitoring, as well as some effective mechanisms for collecting and spending them. On the other hand, the deterrence and effects of green taxes must be thus evaluated. It is thus essential to increase the costs of violations, abuses, and chaos. Designing green taxes and observing economic and political considerations can be accordingly among the decisive factors in the successful implementation of tax policies. There were also several investigations on the effects of green taxes, as mentioned below, whose results were in line with the findings of the present study.

In Hosseinzadeh and Madah [64], the significant effect of green taxes on reduced consumption of environmentally polluting goods had given importance to tax tools as a significant solution to diminish pollution. In Izadkhasti et al. [65], green taxes had also reduced the emission of pollutants. At the same time, minimizing the emission of pollutants had increased health indicators.

The study by Dissou and Eyland [66] had correspondingly stated that the reason for the discrepancy in the results and the effects of pollution taxes in different countries could be attributed to the costs imposed by the implementation of policies on different parts of those countries. Hadian and Ostadzad [67] had further shown that pollution taxes could boost social welfare by reducing the emission of environmental pollutants and demote social welfare by decreasing production and consequently lowering consumption. The results of these two effects could accordingly increase or decrease social welfare [67]. A study in Spain (2011) had also shown that green tax reform was an important tool for environmental protection and could make the tax system much more efficient [68].

In a study (2014), it had been reported that the profitability of polluting industrial products was far and significantly higher than this amount in clean industries. In terms of policies, it was necessary to establish some measures to prevent environmental degradation and health threats by applying green taxes [69]. In another survey (2016), it had been further emphasized that policymakers and planners needed to pay more attention to the appropriate collection of green taxes to achieve sustainable development in order to compensate for environmental degradation [70], which was consistent with the results of the present study.

Another important issue is that for reasons such as increasing attention and improving the effectiveness of tax policies, green tax is better separated from VATs and considered as a separate tax base. Also, Dealing with pollution and environmental damage, must be continuous and decisive. In addition to helping industries to purchase the necessary facilities and modernize equipment, it is essential to increase the cost of violations and lawlessness.

Although health taxes have been mainly studied in high-income countries (HICs), these experiences can be applied for low- and middle-income countries (LMICs), because universal health coverage strategies have become increasingly necessary, and this issue depends on the availability of sustainable financial resources [71].

Considering that in recent years, many countries have started using specific taxes, and at the same time, few studies have been conducted in the field of specific health taxes [40], thus, the findings of this study can also be used in other countries. On the other hand, many topics of this study have been examined according to health conditions in the world and studies conducted in other countries. High consumption of harmful goods such as unhealthy foods and drinks, and smoking, the spread of environmental pollution in the world, followed by health threats, as well as increased attention to taxes on harmful goods and measures, VATs, and green taxes, these are the important and common issues in most countries. Therefore, the results of this study can be generalized to other countries, especially developing countries.

### Recommendations

According to the results of this study, the following suggestions are presented:

- Reforms in the decision-making system, the planning and implementation of related specific taxes, and paying attention to the deterrence of laws and their enforcement guarantee
- Cooperation and coordination by all relevant organizations and ministries in formulating and implementing health-related tax laws (In particular the Ministry of Health and Medical Education, Ministry of Industry, Mine and Trade, Ministry of Agriculture Jihad, Department of Environment, and Municipalities)
- Creating a database and related data on the activity of all industries and manufacturing units, as well as goods, services and actions that affect health, and the accurate determination of examples along with the classification and grading of harmful goods and measures with impacts on health
- building a culture, and raising public awareness of goods, services and actions that are harmful to health, as well as modifying consumption patterns
- Simultaneous and fair attention to all sectors related to health and the accurate and rational allocation of specific health taxes to all relevant organizations and ministries
- Tax exemption or discount to industries and units that produce healthy products, as well as investment in healthy industries and equipment modernization
- Proper and purposeful planning for the correct use of tax resources, transparency in specifying where tax resources can be spent and the close monitoring and follow-up of consumption resources
- Sufficient attention to all types of harmful goods and measures and conducting careful studies on the extent and severity of the effects of these goods and measures on health, and providing practical solutions to reduce consumption or exposure to them
- Pay more attention to the issue of environmental pollution and damage and their impact on people’s health, and subsequent more attention to the important subject of green taxes and its positive effects on public health

### Strengths and limitations

The strength of this study was mainly interviews with policy makers, managers and experts from different organizations and ministries and professors of important universities. The study of health taxes from various aspects, as well as, the effects and relationship of these taxes with the goals and functions of the healthcare sector, were other strengths of this research. Conducting interviews in Farsi (the local language of the interviewees), and translating it into English after transcription, classification and coding, is another strength of the study. The most important limitations of the present study, were the concurrence of the research with the coronavirus epidemic, especially the limitations for face-to-face interviews, the extent of the subject and possibly the inadequate survey of some aspects, as well as, the lack of sufficient studies on various aspects health related taxes.

## Conclusion

Despite the existing limitations and problems, the collection of specific taxes for the health system within the framework of the necessary rules and policies could be pursued more seriously, and this could at least have two benefits, including providing, maintaining, and promoting health along with financing the health system in a sustainable manner. Many strategies to improve public health accordingly need to be legislated, planned, implemented, and monitored properly, especially in the field of financing and enhancing approaches to health, and specific taxes in the health sector could be the most important requirements and facilitators in this regard.

## Data Availability

Since the present study is a qualitative study, based on the statements of the participants in Section of the informed consent form, the participants only allowed the researchers to publish the findings as a whole. Therefore, we do not have permission to provide research data and materials in public or in an appendix. However, the research team is committed to providing some data if requested by other researchers by email to the first author (mahdi19205@gmail.com) or corresponding author (hdargahi@sina.tums.ac.ir).

## Abbreviations

VATs: Value-added taxes
INTA: Iran National Tax Administration,
MD: Medical doctorate
MPs: Members of the parliament
WHO: World health organization
FS: Fiscal space
LMICs: Low- and middle income countries
HICs: High-income countries
